# HIV-1 Vif: a guardian of the virus that opens up a new era in the research field of restriction factors

**DOI:** 10.3389/fmicb.2013.00034

**Published:** 2013-02-20

**Authors:** Akifumi Takaori-Kondo, Keisuke Shindo

**Affiliations:** Department of Hematology and Oncology, Graduate School of Medicine, Kyoto UniversityKyoto, Japan

**Keywords:** HIV-1 Vif, restriction factor, ubiquitin ligase, cell cycle arrest, p53, MDM2

## Abstract

The research on virion infectivity factor (Vif) protein had started in late 1980s right after HIV-1 was cloned, and the function of Vif had been a mystery for a long time. However, the research on Vif has finally lead to the identification of APOBEC3G, which opens up a new era in the research field of host restriction factors in HIV-1 infection followed by TRIM5α, Tetherin/BST-2, and SAMHD1. This suggests that continuation of basic research on fundamental questions is quite important. We still have many questions on Vif and APOBEC3 and should continue to work on these proteins in the future in order to better regulate HIV-1. We will discuss not only the history but also recent advances in Vif research.

## INTRODUCTION

HIV-1 virion infectivity factor (Vif) was identified as an accessory gene right after the HIV-1 genome was sequenced. It is well conserved among lentiviruses except in equine infectious virus and plays a crucial role in the viral life cycle to facilitate viral infectivity as its name indicates (Desrosiers et al., 1998). In the early reports, Strebel and collegues described that the mutant virus deficient in the *vif* gene produces virion particles normally; however, the particles are ~1000 times less infectious than the wild type (Fisher et al., 1987; Strebel et al., 1987). The underlying mechanism of Vif function had been unsolved and a mystery for a long time.

## EARLY OBSERVATIONS OF Vif FUNCTION LEAD TO IDENTIFICATION OF APOBEC3G

Virion infectivity factor exerts its function in a cell-type-specific manner. Vif is dispensable for producing infectious viral particles in permissive cells such as all known adherent cells (e.g., HeLa and 293T cells) and some T cell lines (e.g., CEM-SS and SupT1 cells); in contrast, Vif is indispensable in non-permissive cells such as physiologically relevant CD4^+^ T cells and macrophages, and other T cell lines (e.g., CEM and H9 cells; Gabuzda et al., 1992; Sakai et al., 1993; Simon et al., 1998b). These findings raise two possibilities; one is that permissive cells have a vif-like cellular factor which facilitates virion infectivity, another is that non-permissive cells possess an anti-HIV-1 host factor which is antagonized by Vif. Later studies using heterokaryon experiments have shown the latter possibility (Madani and Kabat, 1998; Simon et al., 1998a). In 2002, Malim’s group identified this factor using very sophisticated subtraction cloning methods between non-permissive CEM cells and its derivative subclone permissive CEM-SS cells, which was first called as CEM15 and is now known as APOBEC3G (Sheehy et al., 2002). Details of functions of APOBEC3G and other APOBEC3 family members are described and discussed in many reviews and other chapters of this issue (Goila-Gaur and Strebel, 2008; Wissing et al., 2010; Kitamura et al., 2011).

In addition to the above described main function, early studies also revealed several important Vif functions including dimerization (Yang et al., 2001), virion incorporation (Camaur and Trono, 1996; Simon et al., 1997), and phosphorylation (Yang et al., 1996; Yang and Gabuzda, 1998); however, the significances of these functions are not discussed much recently. Recently, a novel Vif function on cell cycle has been reported, which is discussed in more detail later.

## Vif ANTAGONIZES APOBEC3G

As described above, the main function of Vif is to antagonize APOBEC3G. Right after identification of APOBEC3G, many studies have shown that Vif inhibits the virion incorporation of APOBEC3G, which is mainly attributable to degradation of cellular APOBEC3G via the proteasomal pathway (Marin et al., 2003; Sheehy et al., 2003; Stopak et al., 2003; Mehle et al., 2004b). However, some studies have also shown that Vif directly inhibits the virion incorporation of APOBEC3G (Opi et al., 2007) or that Vif inhibits translation of APOBEC3G (Mariani et al., 2003; Stopak et al., 2003).

Yu et al. (2003) have independently shown that Vif forms E3 ligase complexes with cellular proteins including Cullin 5, Elongin B, and C (Vif–Cul5–EloB/C complex) using mass-spectrometry techniques. They and others have also shown that this complex works as the E3 ligase for APOBEC3G to induce polyubiquitination of APOBEC3G and direct it to the 26S proteasome for degradation (Mehle et al., 2004a; Yu et al., 2004; Kobayashi et al., 2005). Iwatani et al. (2009) have identified four critical lysine residues (K^297^, K^301^, K^303^, and K^334^) in APOBEC3G which are required for Vif-mediated degradation, although others have reported that Vif can ubiquitinate and degrade a lysine-free APOBEC3G (Shao et al., 2010). Vif also antagonizes other APOBEC3 proteins from APOBEC3C to H by the same E3 ligase complex (Shirakawa et al., 2006).

**FIGURE 1:**
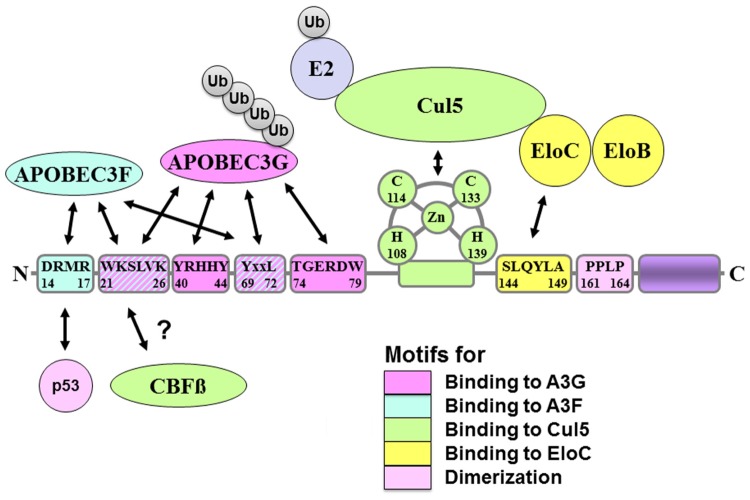
**Schematic figure of the virion infectivity factor (Vif) protein and amino acid motifs for binding to Vif-interacting proteins**. Pink indicates binding motifs for A3G; light blue indicates binding motifs for A3F; light green indicates binding motifs for Cul5; yellow indicates binding motifs for EloC; light pink indicates motifs for dimerization. Vif binds to p53 and CBFβ in its N-terminal regions, but binding motifs were not elucidated yet.

Virion infectivity factor binds to the E3 ligase complex through two interaction sites; it binds to Elongin C through its suppressors of cytokine signaling (SOCS) box motif (Mehle et al., 2004a; Yu et al., 2004), S^144^LQYLA^149^, and to Cullin 5 through a zinc-binding motif (Luo et al., 2005; Mehle et al., 2006), H^108^x_5_Cx_17-18_Cx_3-5_H^139^ (**Figure 1**). The SOCS box motif is well conserved among Vif proteins, indicating that this motif is crucial for Vif function, and mutation of S^144^, a presumed phosphorylation site in Vif, affects binding of Vif to Elongin C (Mehle et al., 2004a). The zinc-binding motif is also important for Vif function to form the E3 ligase complex. Therefore, a zinc chelating agent can inhibit Vif function in infectivity assays (Xiao et al., 2007).

## THE INTERACTION OF Vif WITH APOBEC3 PROTEINS

It is quite important to reveal the interaction sites between Vif and APOBEC3 proteins, because the regulation of this interaction may lead to the development of novel therapeutic strategies for HIV-1 infection. However, their structural information is not fully elucidated yet, because it is quite difficult to produce these proteins as soluble forms. Thus, the information described below is mainly obtained by many studies using site-directed mutagenesis, which sometimes shows different results.

First of all, the most important and confirmed evidence is that the interaction between Vif and APOBEC3G is critically dependent on D^128^PD^130^ in APOBEC3G (Huthoff and Malim, 2007). Many groups have simultaneously reported this evidence by comparing human and African green monkey (agm) APOBEC3G (Bogerd et al., 2004; Mangeat et al., 2004; Schrofelbauer et al., 2004; Xu et al., 2004). In detail, HIV-1 Vif binds and antagonizes human APOBEC3G, but not agm APOBEC3G. In contrast, SIVagm Vif antagonizes agm APOBEC3G, but not human APOBEC3G. By comparing amino acids residues and preparing chimeric APOBEC3G between human and agm APOBEC3G, they identified D^128^ as the determinant of the species-specific binding of Vif to APOBEC3G (Bogerd et al., 2004; Mangeat et al., 2004; Schrofelbauer et al., 2004; Xu et al., 2004). On the other hand, SIVmac and HIV-2 Vif can antagonize both human and agm APOBEC3G, indicating that the interaction between Vif and APOBEC3G is not restricted by D^128^, in other words, D^128^ is not the sole determinant for species-specific target by Vif (Gaur and Strebel, 2012). Furthermore, the interaction between Vif and APOBEC3G is regulated by phosphorylation of APOBEC3G at T^32^ by protein kinase A (Shirakawa et al., 2008).

The interaction sites in Vif are reported by many groups and are much more complicated. The binding site only for APOBEC3G is Y^40^RHHY^44^ (Russell and Pathak, 2007), while that only for APOBEC3F is D^14^RMR^17^ (Russell and Pathak, 2007), and T^74^GERxW^79^ (He et al., 2008). The binding sites for both APOBEC3G and F are W^21^KSLVK^26^ (Chen et al., 2009; Dang et al., 2009), V^55^xIPLx_4-5_LxΦx_2_YWxL^72^ (He et al., 2008), and Y^69^xxL^72^ (Pery et al., 2009; **Figure 1**). To identify the real interaction sites, we have to wait a little longer until we will get the structural information of these complexes.

## Vif AND CBFβ

Recent mass-spectrometry screening of Vif-binding proteins has identified a T cell transcription factor, core-binding factor subunit beta (CBFβ), as an important Vif-binding protein (Jager et al., 2012; Zhang et al., 2012). CBFβ directly binds to Vif and plays a crucial role in forming a stable Vif–Cul5–EloB/C E3 ligase complex. Without CBFβ, the Vif–Cul5–EloB/C E3 ligase complex is not stable enough to polyubiquitinate APOBEC3G and its function is severely impaired. The binding sites of Vif with CBFβ are identified as W^21^ and W^3^^8^ (**Figure 1**). However, the mechanisms by which CBFβ regulates the E3 ligase complex are still under investigation. Furthermore, since CBFβ is an important T cell transcription factor, it would be very interesting to determine whether Vif affects T cell differentiation.

## Vif IS ALSO UBIQUITINATED

**FIGURE 2:**
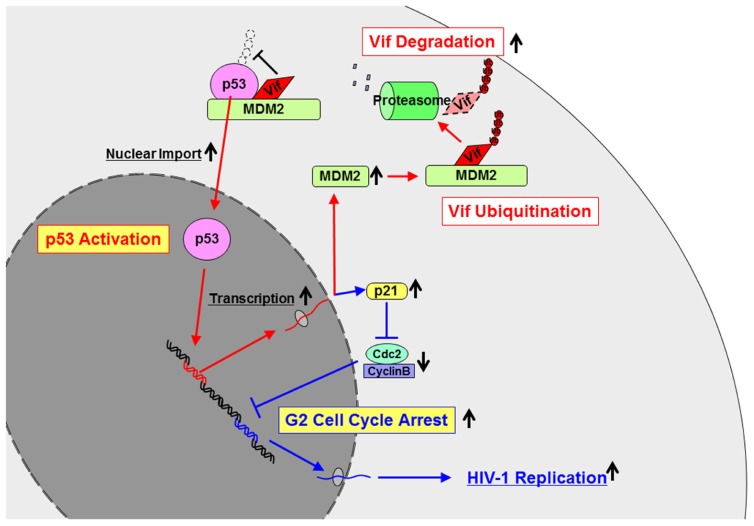
**The mechanisms how Vif is ubiquitinated and degraded and how Vif induces G2 cell cycle arrest**. Vif is ubiquitinated and degraded by MDM2. On the contrary, Vif inhibits ubiquitination of p53 by MDM2 to induce activation and nuclear import of p53. Activated p53 induces transcription of several genes including MDM2 and p21. Enhanced expression of MDM2 may lead to more Vif ubiquitination and degradation, which forms the autoregulatory circuit of Vif expression. On the other hand, activation of p21 leads to G2 cell cycle arrest, resulting in more HIV-1 replication.

Fujita et al. (2004) have reported that expression of the Vif protein in virus-producing cells is maintained at very low levels, which is regulated by the ubiquitin–proteasome pathway. It is because its high expression inhibits viral infectivity by affecting proteolytic processing of Gag protein (Akari et al., 2004). We have identified the E3 ligase for Vif as mouse double minute 2 homolog (MDM2; Izumi et al., 2009; **Figure 2**). Since Vif is a component of a Cul5–EloB/C complex, one report showed that this complex ubiquitinated Vif (Mehle et al., 2004a). Another report showed that other E3 ligases such as neural precursor cell expressed developmentally down-regulated protein 4 (Nedd4) and atrophin-interacting protein 4 (AIP4) bound to Vif, however, it didn’t show the direct evidence of Vif ubiquitination by these ligases (Dussart et al., 2004). The identification of the E3 ligase has lead to elucidation of the mechanisms of Vif-induced G2 cell cycle arrest described below.

## A NOVEL Vif FUNCTION: G2 CELL CYCLE ARREST

In early 1990s, viral protein R (Vpr) had been shown to induce G2 cell cycle arrest in HIV-1-infected cells (He et al., 1995; Re et al., 1995; Roshal et al., 2003; Nakai-Murakami et al., 2006). Many groups have extensively worked on Vpr-induced G2 arrest in terms of its molecular mechanisms and published many papers. Although only one paper reported the virological significance of G2 arrest induced by Vpr (Goh et al., 1998), the basic and fundamental questions of why the virus needs to induce G2 arrest still remain unsolved. More than 10 years had passed since then, and two recent reports came out, describing that Vif as well as Vpr-induce G2 arrest in HIV-1-infected cells (Sakai et al., 2006; Wang et al., 2007). We have recently shown the molecular mechanisms by which Vif induces G2 arrest (Izumi et al., 2010; **Figure 2**). Vif activates p53, which is well known as a tumor suppressor gene and the regulator of cell cycle as “a guardian of the genome.” Vif binds and activates p53 by stabilizing and sequestering it to the nucleus. Activation of p53 induces its downstream cascade such as activation of p21 and inactivation of Cdc2/CyclinB, resulting in G2 arrest. Furthermore, we identified the amino acid residues in Vif responsible for its interaction with p53 and a Vif mutant which does not induce G2 arrest. Using a mutant virus which possesses the *vif* mutant, we have demonstrated that Vif-induced G2 arrest facilitates viral replication (Izumi et al., 2010; **Figure 2**). Thus, HIV-1 needs to have G2 cell cycle arrest to efficiently replicate so that it possesses two accessory genes such as *vif* and *vpr*. Vif induces G2 arrest in a p53-dependent manner, while Vpr accomplishes the same goal in a p53-independent manner.

## CONCLUSION

HIV Vif is an intriguing viral protein, not only because it opens up a new era in the research field of host restriction factors, but also because it has a variety of functions for the viral life cycle by interacting several cellular proteins. It suggests that it might be a good target for control of HIV-1 infection.

## Conflict of Interest Statement

The authors declare that the research was conducted in the absence of any commercial or financial relationships that could be construed as a potential conflict of interest.
